# Reversing the magnetization of 50-nm-wide ferromagnets by ultrashort magnons in thin-film yttrium iron garnet†

**DOI:** 10.1039/d4nh00095a

**Published:** 2024-08-13

**Authors:** Shreyas S. Joglekar, Korbinian Baumgaertl, Andrea Mucchietto, Francis Berger, Dirk Grundler

**Affiliations:** a Laboratory of Nanoscale Magnetic Materials and Magnonics, Institute of Materials (IMX), Ecole Polytechnique Fédérale de Lausanne (EPFL) 1015 Lausanne Switzerland dirk.grundler@epfl.ch; b Institute of Electrical and Micro Engineering (IEM), EPFL 1015 Lausanne Switzerland

## Abstract

Spin waves (magnons) can enable neuromorphic computing by which one aims at overcoming limitations inherent to conventional electronics and the von Neumann architecture. Encoding magnon signal by reversing magnetization of a nanomagnetic memory bit is pivotal to realize such novel computing schemes efficiently. A magnonic neural network was recently proposed consisting of differently configured nanomagnets that control nonlinear magnon interference in an underlying yttrium iron garnet (YIG) film [Papp *et al.*, *Nat. Commun.*, 2021, **12**, 6422]. In this study, we explore the nonvolatile encoding of magnon signals by switching the magnetization of periodic and aperiodic arrays (gratings) of Ni_81_Fe_19_ (Py) nanostripes with widths *w* between 50 nm and 200 nm. Integrating 50-nm-wide nanostripes with a coplanar waveguide, we excited magnons having a wavelength *λ* of ≈100 nm. At a small spin-precessional power of 11 nW, these ultrashort magnons switch the magnetization of 50-nm-wide Py nanostripes after they have propagated over 25 μm in YIG in an applied field. We also demonstrate the magnetization reversal of nanostripes patterned in an aperiodic sequence. We thereby show that the magnon-induced reversal happens regardless of the width and periodicity of the nanostripe gratings. Our study enlarges substantially the parameter regime for magnon-induced nanomagnet reversal on YIG and is important for realizing in-memory computing paradigms making use of magnons with ultrashort wavelengths at low power consumption.

## Abstract 2

Propagating magnon signals stored in periodic and aperiodically patterned ferromagnets with widths down to 50 nm.

## Graphical abstract

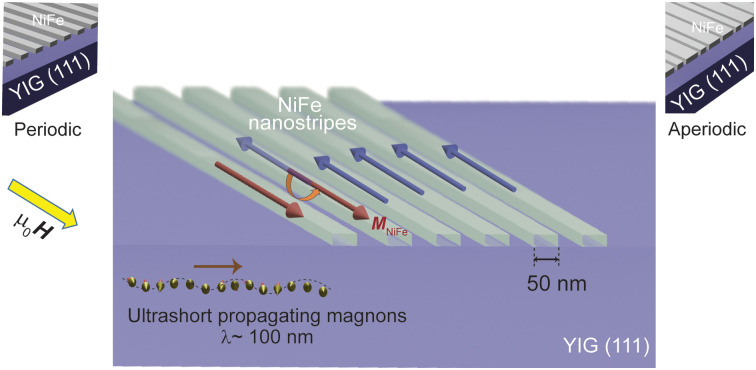

**New concepts**

Propagating spin waves (magnons) in a ferrimagnetic yttrium iron garnet (YIG) film were recently demonstrated to reverse the magnetization of periodically arranged ferromagnetic nanostripes. It is a charge-free alternative of storing GHz signals from wave-based computation in nonvolatile memory bits. However, ultrashort magnons have not yet been explored. We fabricated periodic lattices of 50-nm-wide ferromagnets on YIG and integrated coplanar waveguides for applying microwave signals. They enabled us to directly excite magnons at low power with wavelengths of 100 nm in YIG. We observe that they switch the magnetization of 50-nm-wide ferromagnetic nanostripes after propagating over 25 μm in YIG. Another novel aspect of our work addresses magnon-induced reversal in aperiodically arranged nanomagnets which avoid commensurability effects between magnons and magnetic storage bits of previous experiments. We patterned ferromagnetic nanostripes on YIG exploiting an aperiodic Fibonacci sequence. They gave rise to unexplored magnon branches. Also in these irregular hybrid structures we observe magnon-induced reversal at similar power levels. This observation broadens the design rules when conceiving novel layouts for magnon-based in-memory computing schemes.

The use of various Artificial Intelligence platforms like Dall-E2^1^ and ChatGPT^2^ has skyrocketed in recent months. Such platforms exploit machine learning algorithms to generate and store a tremendous amount of data in computers’ processors and physically separated memory units, respectively, leading to enormous data trafficking. This current approach reduces computing performance and provokes Joule heating losses. The drawbacks of the conventional technology motivate the search for alternatives such as in-memory and neuromorphic computing.^3^ These alternatives require two essential components: nonlinearity in signal processing and nonvolatile memory. Recently, magnonic neural networks have been proposed which exploit wave-based signal processing in a ferrimagnetic thin film combined with an array of nanostructured ferromagnets.^4^ Exploiting propagating spin waves (magnons) in yttrium iron garnet (YIG) and their interference underneath nanomagnets, neural network functionality was demonstrated in that the magnetic states of bistable nanomagnets were iteratively modified and programmed.^4–6^

Spin waves are collective excitations of spins that transfer angular momentum in a magnetically ordered material. In a low-damping material like YIG they can propagate over long distances up to the mm length scale.^7^ They possess engineered wavelengths down to a few tens of nanometers when excited on-chip by microwaves at GHz frequencies.^8,9^ The interaction with distributed magnets controls their scattering, phases and interference.^4,10–12^ A similar hybrid system gives rise to the magnonic holographic memory (MHM) in which coherent spin waves read out stored data *via* interference over several unit cells (magnetic bits).^13^ However, for both the magnonic neural network and MHM, it is not yet decided how to reprogram the magnets and encode the data. Beyond applying a global magnetic^14^ or electrical field,^15^ local heating *via* a scanning laser^16^ or the stray field of a moving cantilever of a magnetic force microscope^17^ might be used to reverse nanomagnets. However, magnetization reversal induced by propagating spin waves avoids additional equipment and mechanical motion of components.

Magnetization switching attributed to magnons in antiferromagnetic NiO was reported by Wang *et al.*^18^ and Guo *et al.*^19^ The observations stimulated large interest.^20^ The type and wavelengths of spin waves leading to magnet reversal were not explored however. Baumgaertl *et al.*^21^ demonstrated that spin waves propagating in YIG induced magnetization reversal of 100 nm wide and 25 to 27 μm long permalloy (Ni_81_Fe_19_ or Py) nanostripes in a small magnetic field *H*. The nanostripes were separated from the spin-wave emitting coplanar waveguide (CPW) by a distance of ≥25 μm. The relevant mode had a wavelength *λ* of about 7 μm. It is now timely to investigate the reversal of nanostripes of different widths initiated by spin waves of shorter wavelength. The study of magnetization dynamics in nanostripes on YIG can also benefit the development of magnon-based microwave devices^22^ and magnon transistors.^23^

In this work, we report the observation of magnon-induced reversal of ferromagnetic nanostripes by propagating magnons in ferrimagnetic YIG which are exchange dominated and have a short wavelength *λ* of ≈100 nm. Exchange-dominated magnons exhibit a parabolic dispersion relation ensuring a high group velocity at ultrashort wavelength (see S1, ESI†). They are key for nanoscale magnon-based devices^8^ but their ability to reverse nanomagnets has not yet been explored. We directly excited the exchange magnons using a microwave-to-magnon transducer consisting of a CPW on YIG which incorporated a lattice of ferromagnetic nanostructures^8,9^ (Fig. 1). The narrowest arrays (gratings) were prepared from 50-nm-wide Py nanostripes whose widths were smaller than in ref. 8, 21 and 24. They allowed us to explore the interaction of exchange magnons in YIG with Py nanostripes for magnon-induced magnetization reversal. We find that magnons with *λ* ≈ 100 nm reverse nanostripes at a spin-precessional power *P*_prec_ of 10.8 nW in a small field *H*. The power value is of the same order as the one reported for dipolar spin waves which had a 70 times longer wavelength.^21^ We studied the magnon-induced reversal of nanostripes of widths ranging from 50 nm to 200 nm and compared the reduction of their coercive fields *H*_C_ by magnons. Going beyond the earlier experiments involving periodic nanostripe arrays with all the same lattice constant *a* = 200 nm,^21,24^ we demonstrate the magnon-induced switching of regular lattices with different *a* and in particular an irregular lattice consisting of arbitrarily placed nanostripes. Thereby we avoid a commensurability effect between *λ* and period *a*. The aperiodically positioned nanostripes lead to an irregular dipolar field distribution which is important in neural networks for magnon based computing.^4^ Our experimental results obtained on exchange-dominated magnons, ultra-narrow ferromagnetic stripes and an aperiodic lattice are promising for enhanced functionalities of such networks and the MHMs operating on the nanoscale.

**Fig. 1:**
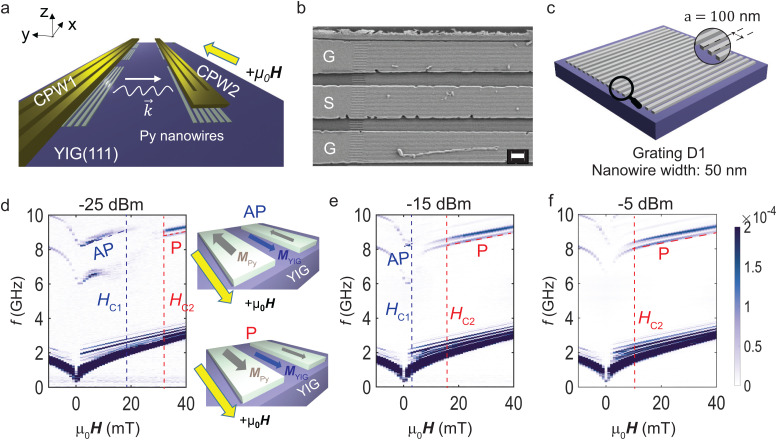
(a) Sketch of the experiment involving Py nanostripes fabricated on 100-nm-thin YIG (111) beneath CPWs separated by a signal-to-signal line distance of 35 μm. (b) SEM image of ground (G) and signal (S) lines of a CPW covering Py nanostripes and the YIG film (sample D1). The lengths of nanostripes alternated between 25 and 27 μm consistent with ref. 21. The scale bar corresponds to 1 μm. The irregular feature on the bottom ground line corresponds to a nonmagnetic residue from the lift-off processing. (c) Illustration of the nanostripe array (grating) shown in (b) with 50-nm-wide stripes and period *a* = 100 nm as indicated in the magnified view. Scattering parameter magnitude of Δ*S*21 measured in transmission configuration between CPW1 and CPW2 using (d) *P*_irr_ = −25 dBm, (e) −15 dBm and (f) −5 dBm. We extract fields *H*_C1_ and *H*_C2_ (indicated by vertical dashed lines) denoting the power-dependent onset and completion, respectively, of the nanostripes’ reversal. The inset in (d) illustrates the relative orientation of magnetization of Py nanostripes (***M***_Py_) and YIG (***M***_YIG_) in AP and P configuration. ***M***_Py_ is anti-parallel (AP) to ***M***_YIG_ and ***H*** in AP configuration (top) and parallel (P) to ***M***_YIG_ and ***H*** in P configuration (bottom).

## Results and discussion

### Magnon mode dependent switching of 50-nm-wide nanostripes

Fig. 1(a) illustrates the experiment. 20-nm-thick Py nanostripes formed gratings on 100-nm-thick YIG grown on a gadolinium gallium garnet GGG (111) substrate. In periodic gratings the stripes exhibited widths *w* of either 50, 100 or 200 nm. The periods amounted to *a* = 2*w*. The sample D1 with stripes of width *w* = 50 nm is shown in Fig. 1(b) and sketched in Fig. 1(c). The period *a* was 100 nm. In addition, we prepared and investigated a sample containing an aperiodic grating. Stimulated by ref. 25, we arranged nanostripes on a Fibonacci sequence. Thereby the stripe positions were not commensurate with a propagating magnon wavelength. The authors of ref. 25 explored the resonances of spin-precessional motion in a Fibonacci sequence prepared from Py stripes positioned on a SiN membrane. They defined reciprocal lattice vectors *G* which we expected to give rise to grating coupler modes if a Fibonacci sequence were applied to YIG under a CPW. CPWs were fabricated above the nanostripes at a signal-to-signal line (edge-to-edge) distance of 35 μm (25 μm). Spin waves were excited at the emitter CPW (CPW1) with port 1 of a vector network analyzer (VNA) and their propagation was analyzed at the detector CPW (CPW2) in that we detected the induced voltages at VNA port 2. An in-plane magnetic field *μ*_0_**H** was applied using current-controlled solenoids. We implemented the following procedures to perform broadband spin-wave spectroscopy at different VNA powers *P*_irr_ between −30 and +15 dBm. The nanostripes were saturated in −*x* direction using *μ*_0_*H* = −90 mT. Spin waves were excited at the given *P*_irr_ for frequencies *f*_irr_ ranging from 10 MHz to 20 GHz with a step of 2.5 MHz. The magnetic field was increased to +40 mT in steps of 1 mT. Transmission spectra *S*21 were recorded at each value *μ*_0_*H*. The median value of the *S*21 spectra measured over the range of magnetic fields was subtracted from the raw data to remove the background signal. Fig. 1(d)–(f) shows the corresponding spectra Δ*S*21 for three different values *P*_irr_ as indicated in the panels. The dark branches correspond to different spin wave modes propagating from CPW1 to CPW2. With increasing power, the high-frequency branches (grating-coupler modes) labelled by P start at smaller and smaller field *H* (red dashed lines). At the same time, the branches AP end at smaller and smaller *H* (purple dashed lines) or do not appear in panel (f). We attribute the P (AP) branches to a magnetic configuration in which Py nanostripes are aligned with (are anti-parallel to) the magnetization of the YIG. YIG reverses its magnetization at a field below 2 mT.

In an intermediate field regime the high frequency branches AP and P are not resolved indicating that the gratings are not uniformly magnetized. In ref. 8 such magnetic configurations of gratings were labelled by the term “random”. The vanishing signals of grating coupler modes indicated the random magnetic orientation of stripes. The field range of the corresponding random regime reflected the distribution of switching fields (coercive fields) of Py nanostripes. Switching field distributions were addressed in, for instance, ref. 8, 21 and 26–30.

Strong transmission signals of high-frequency spin wave modes reappear in P configuration after the magnetization vectors of nanostripes under both CPWs are symmetric again,^31^ in other words, when the nanostripes beneath both CPWs are switched and have regained saturated magnetic order.^8,30^ Following ref. 21, the disappearance and reappearance of high-frequency branches at positive fields define critical field values *H*_C1_ and *H*_C2_, respectively, which quantify the switching field distribution of the nanostripe arrays. The critical field *H*_C1_ (*H*_C2_) is given by the value of applied magnetic field *H* at which the signal strength of the branch AP (P) has decreased (increased) to 50% of its maximum signal strength. The two critical fields are attributed to the onset and completion of nanostripes’ reversal. In sample D1, we extracted *H*_C1_ and *H*_C2_ by analyzing the spin wave mode with wave vector *k* = 2π/*λ* = *k*_1_ + 1*G*^D1^ and *G*^D1^ = 2π/*a* = 2π/(100 nm). *k*_1_ corresponded to the most prominent wave vector provided by the CPW which resided in the dipolar regime of the spin-wave dispersion relation and *G*^D1^ to the reciprocal lattice vector of sample D1. With increasing *P*_irr_, *H*_C1_ and *H*_C2_ decreased [Fig. 1(d)–(f)]. In Fig. 1(f), branch AP was no longer observed. This finding indicated that the reversal of 50-nm-wide nanostripes started at an applied field as low as 1 mT for *P*_irr_ = −5 dBm.

**Fig. 2:**
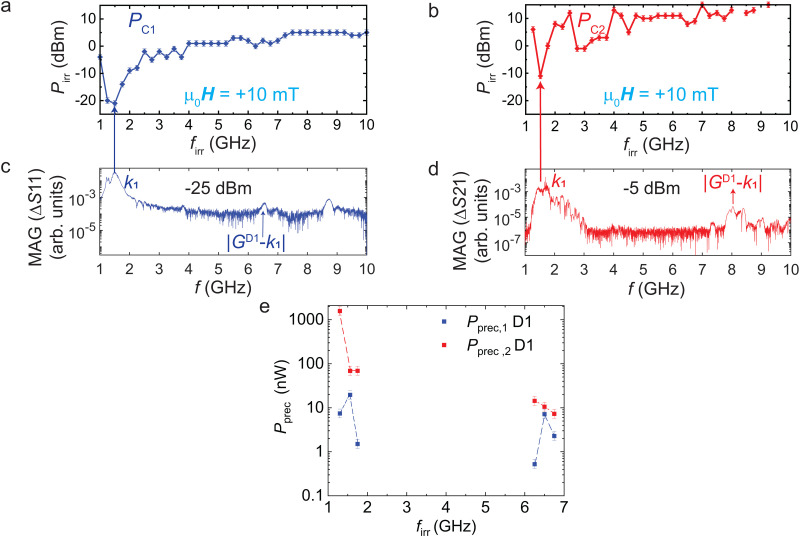
(a) Switching yield diagram of D1. The symbols interconnected by lines indicate the frequency-dependent critical power attributed to the reversal of about 50% of the nanostripes below CPW1 at +10 mT. (b) Magnitude of Δ*S*11 obtained at +10 mT with a low power *P*_sens_ of −25 dBm. (c) Switching yield diagram reflecting nanostripes’ reversal below CPW2. (d) Magnitude of Δ*S*21 measured at +10 mT using *P*_sens_ of −5 dBm. The spectrum differs from (b). A clear shift of mode |*G*^D1^ − *k*_1_| from 6.5 GHz in (b) to 8 GHz in (d) is resolved. For (d), we assume nanostripes beneath both CPWs to be switched by mode *k*_1_ which is excited at a power larger than *P*_C1_ and *P*_C2_ at 1.5 GHz. (e) Precessional power values *P*_prec_ at frequencies corresponding to modes *k*_1_ and |*G*^D1^ − *k*_1_|.

Before discussing the power dependence of critical fields in different samples it is instructive to identify the magnon modes in sample D1 which induce most efficiently nanostripe reversal in a fixed field of +10 mT. This field value is smaller than *μ*_0_*H*_C1_ determined at −30 dBm. For different *f*_irr_ the power *P*_irr_ was increased until the branch AP (P) in Δ*S*21 reduced (increased) to 50% of its maximum signal strength (see Methods and S2, ESI†). These critical power values *P*_C1_ (*P*_C2_) are summarized in the switching yield diagrams of Fig. 2(a) and (c). We find that the mode *k*_1_ (*λ* = 7.2 μm) which is directly excited by CPW1 at 1.5 GHz [Fig. 2(b)] induces reversal of 50-nm-wide Py stripes at a power *P*_C1_ = *P*_irr_ = −20 dBm underneath CPW1. At *P*_C2_ = −11 dBm, mode *k*_1_ [Fig. 2(d)] reversed about 50% of nanostripes underneath CPW2. These observations are qualitatively consistent with ref. 21. For the grating coupler mode with *k* = |*G*^D1^ − *k*_1_| (*λ* = 101.4 nm) the corresponding power values read *P*_C1_ = +2 dBm and *P*_C2_ = +8 dBm, respectively. We note that this magnon mode with ultrashort wavelength propagated over 25 μm through bare YIG and induced nanostripe reversal underneath CPW2. This observation is promising for nanoscale magnonic devices and has not been achieved in ref. 21. We note that at large powers *P*_irr_ reversal is found also at frequency values *f*_irr_ which do not have a one-to-one correspondence with spin wave branches detected phase-coherently by port 2 of the VNA. In ref. 24 it was shown that large excitation powers lead to parametric pumping of spin waves which reside at smaller frequencies than *f*_irr_ and propagate through YIG. These longer-wavelength spin waves are not detected by the VNA setup operated in the linear response regime but are still able to reverse nanostripes. We refer to ref. 24 concerning a detailed discussion.

To compare the efficiency in magnon-induced stripe reversal between different samples, we evaluate the power *P*_prec_ transferred to the spin precession (prec) in YIG.^21^ This parameter is independent of the individual microwave-to-magnon transduction of CPWs. The precessional power values *P*_prec_ are evaluated by multiplying the critical power *P*_C1_ or *P*_C2_ obtained from the switching yield diagrams with the square of the magnitude of Δ*S*11 (see S3, ESI†) measured at −30 dBm and −10 dBm, respectively. Fig. 2(e) summarizes precessional power values *P*_prec,1_ and *P*_prec,2_, respectively, for frequencies at which the magnon modes *k*_1_ (dipolar magnon) and *k* = |*G*^D1^ − *k*_1_| (exchange magnon) are excited. In ESI,† S1, we provide a dispersion relation of YIG thin film plotted at +10 mT. We observe that the mode *k*_1_ possesses *P*_prec,2_ of 68.9 nW at the emitter CPW when reversing nanostripes remotely positioned at the detector CPW. This value is similar to the one found in ref. 21. Strikingly, the grating coupler mode |*G*^D1^ − *k*_1_| with an ultrashort wavelength of 100 nm requires *P*_prec,2_ of only 10.6 nW for reversing the same 50-nm-wide Py stripes. The data demonstrate that ultrashort exchange magnon modes are more efficient than long-wavelength dipolar modes in terms of spin-precessional power needed for reversal after propagating 25 μm in YIG. Our observations support the concept of dynamic dipolar coupling^23^ and its enhancement suggested in ref. 24 which proposes efficient reversal for a magnon's wavelength *λ* being twice the nanostripe's width *w*.

### Magnon-induced reversal of nanostripes in an aperiodic lattice

**Fig. 3:**
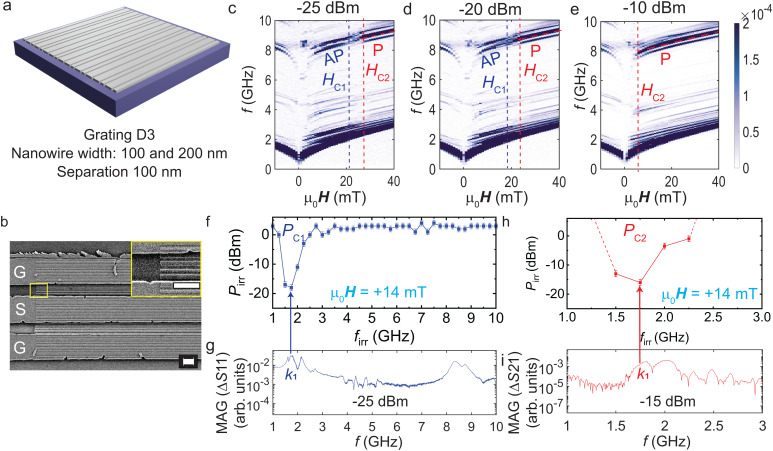
(a) Schematic of sample D3 with a Fibonacci grating consisting 100-nm- and 200-nm-wide Py nanowires separated by 100 nm. (b) SEM micrograph of sample D3 showing Py nanostripes patterned in a Fibonacci sequence under a CPW and in its gaps between ground (G) and signal (S) lines. The inset shows a magnified view of the nanostripes. Scale bars correspond to 1 μm. (c)–(e) Plots of magnitude of Δ*S*21 spectra measured for the device at increasing applied microwave powers (*P*_irr_). (f) Switching yield diagram (in blue) showing the critical power that corresponds to switching of the gratings beneath CPW1 by *k*_1_ mode. (g) Magnitude of the reflection spectrum Δ*S*11 measured at +14 mT showing the *k*_1_ mode. (h) Switching yield diagram (in red) of the nanostripes beneath CPW2. Here, we focus on the frequencies around the *k*_1_ mode that is observed in the (i) magnitude of Δ*S*21 measured at +14 mT. Dotted lines in (h) are a guide to the eye. They indicate that the critical power *P*_C2_ for modes at *f*_irr_ < 1.5 GHz and >2.5 GHz is beyond +6 dBm.

In sample D3 nanostripes were arranged in a quasi-crystalline Fibonacci sequence beneath both the CPWs [Fig. 3(a)]. The nanostripes were 100 nm and 200 nm wide separated by a 100-nm-wide air gap. The scanning electron microscopy (SEM) micrograph of the fabricated nanostripe array beneath a CPW is shown in Fig. 3(b).

Fig. 3(c) shows the magnitude of Δ*S*21 measured at *P*_irr_ = −25 dBm. Dark branches correspond to the spin wave modes propagating in YIG from CPW1 to CPW2. Multiple spin wave modes are resolved. They show that the aperiodic lattices function as grating couplers. The high-frequency modes are excited and detected due to reciprocal lattice vectors provided by Fibonacci gratings^25^ beneath the CPWs. Again, these grating coupler modes can be divided into low-field AP branches and high-field P branches. Similar to Fig. 1(c)–(f), these branches vanish and reappear when the applied magnetic field is swept from −30 mT to +40 mT. Branches chosen for the analysis of the nanowires’ switching fields are marked by a dashed purple line (AP mode) for the onset of switching and by a dashed red line (P mode) for the completion of switching. Fig. 3(c)–(e) shows spectra for three different VNA power values *P*_irr_. *H*_C1_ (purple vertical dashed line) and *H*_C2_ (red vertical dashed line) decrease with *P*_irr_. The AP branch approaches zero field for *P*_irr_ above −15 dBm, *i.e.*, at a smaller power compared to D1, but similar to D2 (see below). We attribute this finding to a smaller coercive (shape anisotropy) field of the 200-nm-wide nanostripes compared to the 100-nm-wide ones in the aperiodic array. Reversing wide stripes leads to magnetic disorder and the disappearance of grating coupler modes.

Switching yield experiments were performed on D3 similar to D1 but at a field of +14 mT. The sample D3 was saturated at *μ*_0_*H* = −90 mT and then the field was swept to +14 mT, leaving the nanostripes in the AP configuration. We used intentionally the same field value like in ref. 21 to compare directly with the reversal reported for the periodic grating of 100-nm-wide nanostripes with 100-nm-wide gaps. The spin waves were excited at irradiation frequencies *f*_irr_ ranging from 1 GHz to 10 GHz with a 0.25 GHz step. At each *f*_irr_, the microwave power *P*_irr_ was increased from −25 dBm to +6 dBm. The state of nanostripes was recorded by measuring Δ*S*21 in the frequency window of 2.5 to 5.5 GHz. The critical powers at which branches first reduced and then increased to 50% of the maximum signal strength were denoted as *P*_C1_ and *P*_C2_, respectively. The symbols displayed in Fig. 3(f) and (h) reflect the onset (completion) of the reversal of stripes beneath CPW1 (CPW2). The switching yield diagrams show that mode *k*_1_ induces the reversal of nanostripes below CPW1 at 15.8 μW and below CPW2 at 25.1 μW. The latter value is a factor of two smaller compared to ref. 21 where periodically arranged stripes with *w* = 100 nm were reversed underneath CPW2 by mode *k*_1_ in a field of +14 mT. We note that grating coupler modes in sample D3 did not allow us to reverse nanostripes beneath CPW2 and extract *P*_C2_ up to *P*_irr_ = 8 dBm. This is unlike the observation in case of sample D1 with periodically arranged 50-nm-wide nanowires. We attribute the absence of magnon-induced reversal up to 8 dBm to the reduced excitation strength reported for aperiodic grating couplers.^32^ Thereby the threshold amplitude for reversal was not reached.

### Magnon-induced switching dependent on nanostripe width

**Fig. 4:**
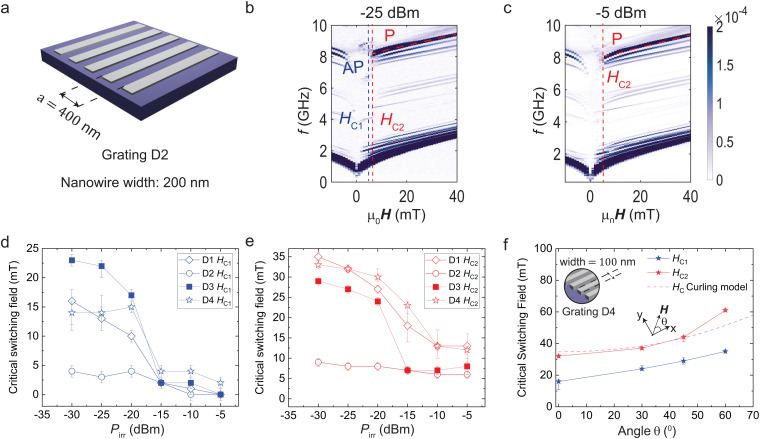
(a) Sketch of sample D2 with periodic gratings consisting of 200-nm-wide Py nanostripes arranged with *a* = 400 nm. Δ*S*21 spectra measured on D2 at a VNA power of (b) −25 dBm and (c) −5 dBm. (d) Critical field *H*_C1_ and (e) *H*_C2_ extracted for nanostripes with widths of 50 nm (D1), 100 nm (D4, taken from ref. 21), and 200 nm (D2) denoted by hollow symbols as well as the aperiodic grating (D3) denoted by solid rectangles which incorporates nanostripes with two different values *w*. (f) Critical switching fields (symbols) extracted from transmission signals measured on sample D4 at various angles *θ* of the applied in-plane field (inset) using *P*_irr_ = −25 dBm. Connecting lines are guides to the eyes. The dashed line represents the calculated coercive field as a function of *θ* assuming reversal *via* curling (see S7, ESI†).

In this section, we compare the critical switching fields for samples incorporating different nanostripe widths. Fig. 4(a)–(c) shows the parameters and spin-wave spectra obtained on sample D2 with *a* = 400 nm and *w* = 200 nm. The switching fields *H*_C1_ and *H*_C2_ reduce with power *P*_irr_. However, their values and variation are much smaller than for D1. In Fig. 4(d) and (e) we summarize power-dependent critical fields extracted for three different samples D1, D2 (hollow symbols), D3 (solid rectangle) and compare them to sample D4 reported in ref. 21 (star). A decreasing trend in *H*_C1_ and *H*_C2_ with *P*_irr_ is observed for all samples substantiating magnon-induced reversal for nanostripes of different widths between 50 and 200 nm.

A detailed analysis provides two key observations:

(1) D1, D3 and D4 show sharp reductions in *H*_C1_ and *H*_C2_ as compared to D2. The net reduction in *H*_C2_ due to spin wave excitation between −25 dBm and −5 dBm in D1, D3 and D4 is about five times larger than in D2 (see S4, ESI†).

(2) In Fig. 4(d) and (e), sample D1 with *w* = 50 nm behaves similarly to D4 with *w* = 100 nm presented in ref. 21 (star). Only D2 with *w* = 200 nm shows critical fields which are much lower at low power than all other samples.

Fig. 4 shows that magnon-induced reversal occurred for both the narrowest nanostripes with a large reversal field of up to about 35 mT and the widest ones with a reversal field of 10 mT and smaller. Topp *et al.* in ref. 26 presented an analytical model allowing one to calculate the effective transverse demagnetization factor *N*_eff_ for nanostripes arranged in periodic lattices. The model quantifies the effect of their dipolar interaction which is known to modify the shape anisotropies.^33^ We find *N*_eff_ = 0.2 for D1, 0.1 for D4 and 0.05 for D2. Assuming coherent rotation of spins, the coercive fields should reflect the different magnetic anisotropy fields *N*_eff_*μ*_0_*M*_S_ of the samples and scale accordingly:^34,35^ one would expect a clearly larger reversal field for D1 on the order of *N*_eff_*μ*_0_*M*_S_ = 0.2 T compared to 0.1 T for D4. We find however, that samples D1 and D4 show smaller and very similar fields *H*_C2_ at the lowest VNA power, inconsistent with a coherent reversal mechanism. If one assumes instead incoherent reversal *via e.g.* curling^36^ or domain wall motion,^37,38^ the energy barrier due to shape anisotropy does not play a role. Instead, the nanostripes reverse at lower fields.

To explore the reversal mechanism, we considered ref. 39–44. We performed VNA measurements on device D4 at low power and studied the reversal of the 100-nm-wide Py nanostripes at an external field *μ*_0_**H** applied along the angle *θ* to their long axis. We excited propagating magnons at *P*_irr_ = −25 dBm, and measured *S*21 spectra at *μ*_0_*H* swept from −90 mT to +90 mT at *θ* = 0°, 30°, 45° and 60°. Identifying the random magnetic orientation as a function of *θ* we extracted critical switching fields *H*_C1_ and *H*_C2_ as shown in Fig. 4(f). The dashed line represents the coercive field expected for reversal by means of curling^39,41^ (see Section S7 of the ESI† for details). The experimentally extracted critical fields follow the predicted trend. We note however that a full quantitative comparison between experiment and model would need the consideration of the underlying YIG and its effect on the spatial distribution of magnetic charges which determine *N*_eff_. The underlayer was not contained in the models outlined in ref. 26, 39 and 41. Still, the small values of experimentally determined coercive (critical) fields and their dependence on *θ* suggest incoherent reversal *via* curling. The reversal mechanism was not discussed in earlier works.^21,24^

Importantly, our experiments showed that Py nanostripes having widths down to 50 nm were reversed by propagating spin waves in YIG whose *λ* differed by a factor of 70 and that a short wavelength-magnon with *λ* = 2*w* was efficient. The presented results extend the findings of ref. 21 and 24 to smaller width *w*, shorter wavelength *λ* and longer propagation length. In case of *λ* ≈ 100 nm, the propagation distance was ≥250 × *λ*. Such a macroscopically large distance allows for both coherent scattering in a nanostructured holographic memory^13^ and computation across several unit cells of a neural network^4^ before inducing nonvolatile storage of the computational result in a separate magnetic bit.

### Conclusion

Power dependent spectroscopy performed on thin YIG containing arrays of Py nanostripes with widths *w* ranging from 50 to 200 nm evidenced their magnon-induced reversal in small magnetic fields. We observed on the one hand a sharp decrease in the reversal fields of stripes with *w* = 50 nm after applying a VNA power of about −20 dBm (10 μW) to the spin-wave emitting coplanar waveguide. The sharp decrease in switching field was observed also in the sample with an aperiodic arrangement of nanostripes with *w* = 100 nm and 200 nm. On the other hand, a more steady decrease of reversal field was seen in case of periodic arrays with 200-nm-wide nanowires. However, their initial reversal field was already small. Spin waves excited directly by the CPW exhibited *λ* ≈ 7 μm and reversed nanostripes in all the four investigated samples. Exchange magnons with *λ* ≈ 100 nm induced efficiently the reversal of the 50-nm-wide Py nanostripes. The required precessional power was as low as 11 nW in a small field of +10 mT. By means of angle-dependent measurements we concluded that curling was the most likely reversal mechanism of the 100-nm-wide Py nanostripes on YIG. Though the microscopic origin of the magnon-induced torques for reversal has not yet been unequivocally explained it is important to highlight that we achieved the switching of Py nanostripes with very different widths by either dipolar or exchange-dominated spin waves which propagated over 25 μm in YIG away from the spin wave emitter. Our findings are important for nanoscale magnonic in-memory computation, the holographic memory and reconfigurable spin wave-based computing devices.

## Methods

### Sample fabrication

We used a 100-nm-thick YIG thin film epitaxially grown on a GGG (111) substrate. It was provided by the Matesy GmbH in Jena, Germany. A 20-nm-thick Py (Ni_81_Fe_19_) film was deposited on the YIG film using electron beam evaporation. Two groups of gratings of nanostripes separated by 25 μm were etched using a resist mask prepared *via* electron beam lithography applied to hydrogen silsesquioxane. The sample D1 (D2) was patterned with 50 nm (200 nm) wide nanostripes periodically arranged in a 100 nm (400 nm) period. The lengths of nanostripes were alternately varied between 25 and 27 μm. D3 was fabricated with 25 μm long 100-nm- and 200-nm-wide nanostripes arranged in Fibonacci sequence with a gap of 100 nm. The samples were etched with an Ar ion beam considering an etch stop at the YIG film. Two coplanar waveguides (CPWs) were patterned above the nanostripe gratings with signal-to-signal line separation of 35 μm using electron beam lithography with an MMA-PMMA double layer positive resist, electron beam evaporation of 5-nm-thick Ti and 110-nm-thick Cu followed by lift-off processing. Ti was deposited for adhesion.

### Broadband spin wave spectroscopy

The magnon-induced reversal was investigated using Keysight Vector Network Analyzers (VNAs) N5222A and N5222B. VNAs were calibrated using SOLT (Short-Open-Load-THRU) calibration to remove the background noise arising from RF components like microwave cables, adapters and GSG microbprobes. A detailed information can be found in the S6 (ESI†). The probe station of the VNA setup consisted of electromagnetic pole shoes which provided a bipolar magnetic field of up to 90 mT at arbitrary in-plane directions. The CPW1 or emitter CPW of the sample was connected to the VNA port 1 with a microprobe. CPW2 is connected to the detector port 2 with an identical microprobe. Initially, the sample was saturated at −90 mT (in −*x* direction) and spin waves were excited with microwaves of frequencies ranging from 10 MHz to 20 GHz with a step of 2.5 MHz. The scattering parameters *S*11 and *S*21 in reflection and transmission configuration, respectively, were recorded. The static field was brought to −30 mT and increased to +40 mT in a step-wise manner. At each step, the scattering parameters were recorded. The measurements were carried out for spin wave modes excited with applied microwave powers from −25 dBm to +6 dBm. These all-electrical spin-wave spectroscopy (AESWS) measurements were repeated for devices with different grating periods and nanostripe widths and also for gratings with nanowires arranged in Fibonacci sequence. After every measurement, the median subtracted magnitude of scattering parameters as a function of static magnetic field were plotted and analyzed for different spin wave modes. The critical switching fields, *i.e.* static magnetic fields at which the gratings started (*H*_C1_) and completed reversal (*H*_C2_) were decided based on the disappearance and reappearance of a specific spin wave branch for all devices between 8–10 GHz at magnetic fields swept up to +40 mT.

### Switching yield diagram

We performed switching yield diagram measurements as follows. A positive opposing field *μ*_0_*H* was applied to a sample after saturation at negative fields. A transmission spectrum *S*21 was measured within a frequency window of 6–9.5 GHz for D1 and 2.5–5.5 GHz for D3 (*f*_sens_) excited at −25 dBm (*P*_sens_). We call this operation as sensing of the nanostripes’ magnetic configuration being either anti-parallel (before) or parallel to the small bias field (after magnon-induced reversal). Spin waves were excited with irradiation frequency (*f*_irr_) range of 1–1.25 GHz at an irradiation power of −25 dBm (*P*_irr_). After the irradiation, a *S*21 transmission spectrum was measured in the sensing window (*f*_sens_) to sense the effect of the irradiation on the nanostripes. This measurement sequence was repeated for the spin wave mode excited at an increasing *P*_irr_ from −25 dBm to the maximum of +6 dBm in case of the D3 and up to +15 dBm in case of the D1. The magnetic field protocol was reset and the measurements were repeated at the next interval of irradiation frequency (*f*_irr_) up to 10 GHz. As an example, median subtracted magnitude Δ*S*21 transmission spectra plotted for D1 measured at *f*_irr_ from 1.75–2 GHz across the range of *P*_irr_ are provided in the S2 (ESI†). The spin wave irradiation powers inducing a disappearance and reappearance of spin wave branches in these plots corresponded to critical powers needed to start (*P*_C1_) and complete (*P*_C2_), respectively, the switching of nanostripes below CPW1 and CPW2, respectively.

## Author contributions

S. S. J., K. B., and D. G. planned the experiments. K. B. and D. G. designed the samples. K. B. prepared the samples. S. S. J. performed the experiments together with K. B., F. B. and A. M. S. S. J., F. B., A. M. and D. G. analyzed and interpreted the data. S. S. J. and D. G. wrote the manuscript. All authors commented on the manuscript.

## Data availability

Data for this article are available at zenodo at [URL – https://doi.org/10.5281/zenodo.13310436]. The data supporting this article have been included as part of the ESI.†

## Conflicts of interest

The authors declare no conflict of interest.

## Supplementary Material

NH-009-D4NH00095A-s001
