# Supplementary material for: Reversing the magnetization of 50-nm-wide ferromagnets by ultrashort magnons in thin-film yttrium iron garnet
Source: Nanoscale Horiz. 2024 Aug 13;9(10):1740–8. doi: 10.1039/d4nh00095a (PMC11339637; doi:10.1039/d4nh00095a)
Supplement: NH-009-D4NH00095A-s001 [file NH-009-D4NH00095A-s001.pdf]

Supplementary Information (SI) for Nanoscale Horizons.
This journal is © The Royal Society of Chemistry 2024

Supporting Information

Reversing the magnetization of 50-nm-wide ferromagnets by ultrashort magnons in thin-film Yttrium Iron Garnet

Shreyas S. Joglekar,[†,¶] Korbinian Baumgaertl,[†,¶] Andrea Mucchietto,[†] Francis Berger,[†] and Dirk Grundler[*,†,‡]

†Ecole Polytechnique Fédérale de Lausanne (EPFL), Institute of Materials, Laboratory of Nanoscale Magnetic Materials and Magnonics, CH-1015 Lausanne, Switzerland

‡Institute of Electrical and Micro Engineering, CH-1015 Lausanne, Switzerland

¶ Equal contribution

E-mail: dirk.grundler@epfl.ch

1. Dispersion relation of surface spin waves for a 100-nm-thick YIG film at an applied field of +10mT

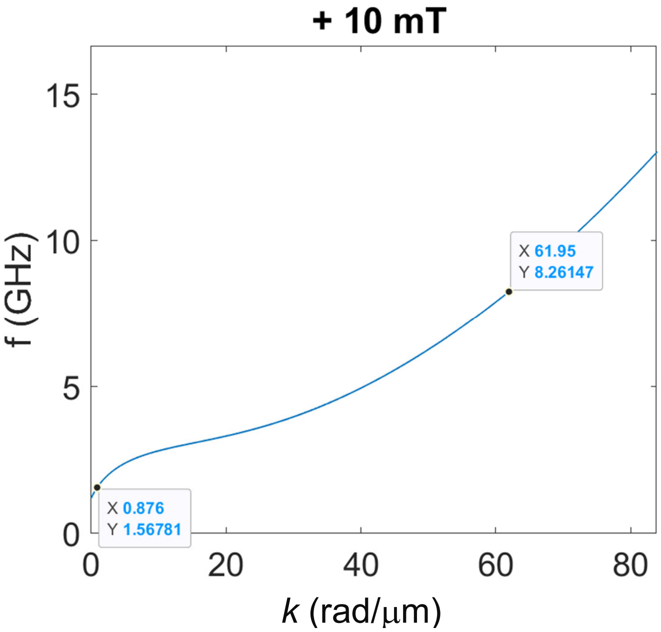

Figure S1: Dispersion relation of spin waves calculated for a 100-nm-thick YIG film at +10 mT (Damon-Eshbach configuration) using the Kalinikos and Slavin formalism. The right box indicates the wavevector *k* of the first grating coupler mode (X-value given in rad/μm) excited by a one-dimensional lattice of 50-nm-wide ferromagnetic nanostripes. The wavevector lies in the exchange-dominated regime with a parabolic relation between *f* and *k*.

2. **Criteria for extraction of critical powers P_{C1} and P_{C2} from switching yield diagram experiment for device D1**

The switching yield diagram is a plot of critical power needed to start and complete the switching of gratings beneath both the CPWs. This is calculated with the following strategy:

- The device is saturated at -90 mT and the applied magnetic field is swept up to +10 mT (for D1). Median subtracted magnitude of Δ*S*21 is measured at P_{sens} =-25 dBm and in a specific frequency window (f_{sens}: 6 to 9.5 GHz), where the magnon modes are detected (black branches in figure S2a). Then, a specific magnon mode is excited at f_{irr} (here, 1.75 to 2 GHz) and for P_{irr} stepped from -25 dBm to +15 dBm. After every irradiation step P_{irr}, the magnitude of Δ*S*21 is measured at P_{sens} =-25 dBm in the frequency window f_{sens}.
- The criteria of extracting P_{C1} and P_{C2} are given by the disappearance of magnon modes and reappearance at a new frequency highlighted with blue and yellow brackets.

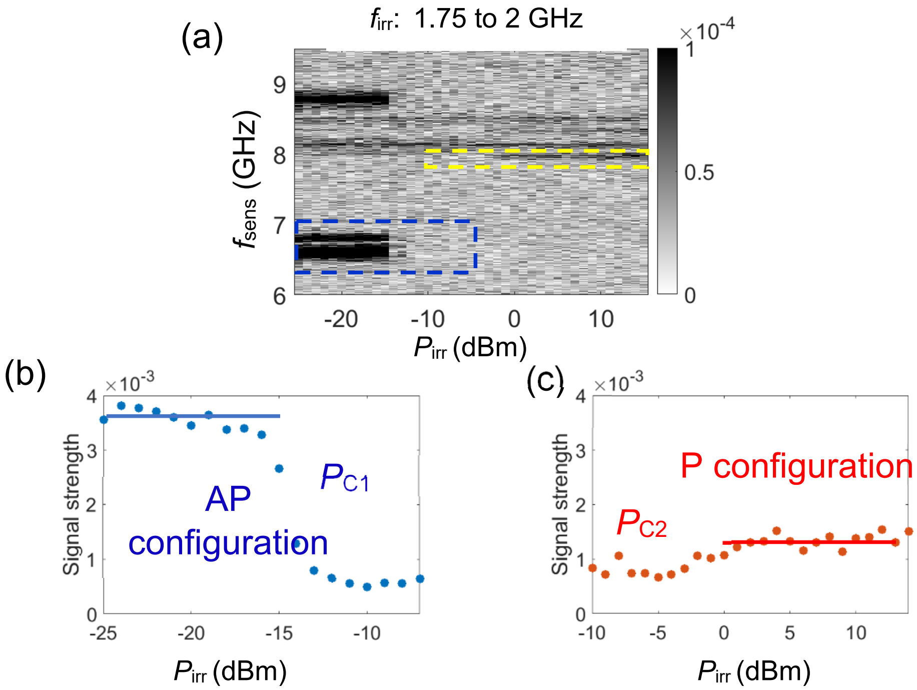

Figure S2: Procedure for determining the critical switching powers (P_{C1}) and (P_{C2}) from the *S*21 transmission data after exciting a magnon mode from 1.75 to 2 GHz at an increasing irradiation power (P_{irr}). The two black branches between 6.5 and 7 GHz as well as 8.5 and 9 GHz correspond to the spin wave modes sensed for P_{irr} below ~ -15 dBm. The integrated signal strength from f_{sens} between 6.4 to 7 GHz (marked with blue dotted lines) was extracted and plotted as a function of P_{irr} in (b) showing the disappearance of the mode between P_{irr} = -25 dBm and -10 dBm. P_{C1} corresponds to P_{irr} at which the mode is at 50% of its maximum signal strength with respect to the minimum signal strength or noise floor. For the given *S*21 spectra, it is -14 dBm with an error bar of ± 1 dBm. On the contrary, a spin wave mode appears at 8 GHz for P_{irr} > 0 dBm. We apply a similar criterion but now for the increase in the signal strength for the branch highlighted in yellow dotted lines. The signal strength was extracted between 7.8 to 8.04 GHz and plotted in (c) as a function of P_{irr}. The critical switching power at which a new mode appears is denoted as P_{C2} and is given by 50% of maximum signal strength of the branch. Here, it corresponds to 0 dBm with an error bar of ± 1 dBm.

3. Calculation of precessional power (P_{prec}) at P_{C1} and P_{C2} for device D1

The precessional power for a magnon mode with frequency f_{irr} excited at microwave power (P_{irr}) is given by:

$$P_{prec} = (\mathrm{MAG}(\Delta S11) \text{ at } f_{irr})^2 \times (P_{irr}),$$

where, P_{C1} and P_{C2} are considered as P_{irr} and obtained from a switching yield diagram measurement as shown in the previous section.

$$\mathrm{MAG}(\Delta S11) = \mathrm{MAG}(S11)^{\text{at 10 mT}} - \mathrm{MAG}(S11)^{\text{at 0 mT}}$$

The spectra $S11$ measured at 0 mT is subtracted from the Raw spectra measured at 10 mT to reduce the background signal.

The following plot shows reference subtracted $\Delta S11$ spectra.

$P_{prec,1}$ was calculated with $S11$ spectra measured at -30 dBm, where, all gratings are antiparallel to YIG and the applied field direction. For $P_{prec,2}$, we use the spectra measured at -10 dBm. At this microwave power the gratings are completely switched beneath both the CPWs.

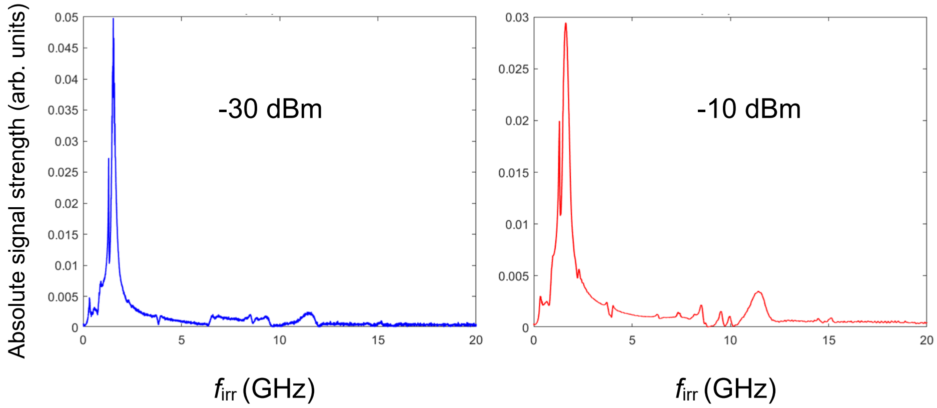

Figure S3: Magnitude of $\Delta S11$ spectra at 10 mT measured at -30 dBm and -10 dBm after subtracting the reference spectrum taken at 0 mT. The signal strength for both k_1 and $|G^{D1} - k_1|$ modes were extracted and used to evaluate $P_{prec,1}$ and $P_{prec,2}$

4. Critical field reduction extracted from *S*21 spectra of devices D1 (50 nm wide nanostripes) and D2 (200 nm wide nanostripes) measured at different P_{irr}

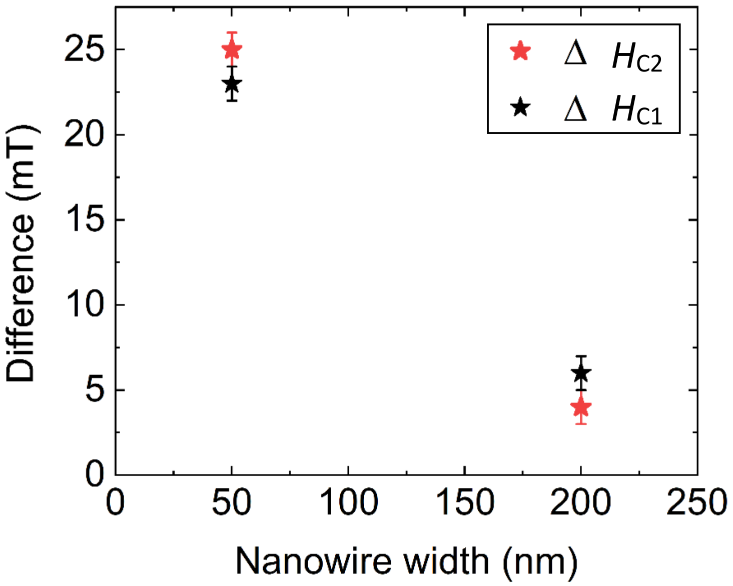

Figure S4: Difference in critical switching fields extracted for the devices D1 and D2 consisting of 50-nm-wide and 200-nm-wide Py nanostripes, respectively. The data points correspond to the difference between H_{C1} and H_{C2} values extracted from Δ*S*21 spectra measured at -30 dBm and -5 dBm (Fig. 4 (d) and (e) in the main text).

5. **Onset of branch P near zero field for device D2 at -5 dBm (showing absence of a branch corresponding to AP configuration)**

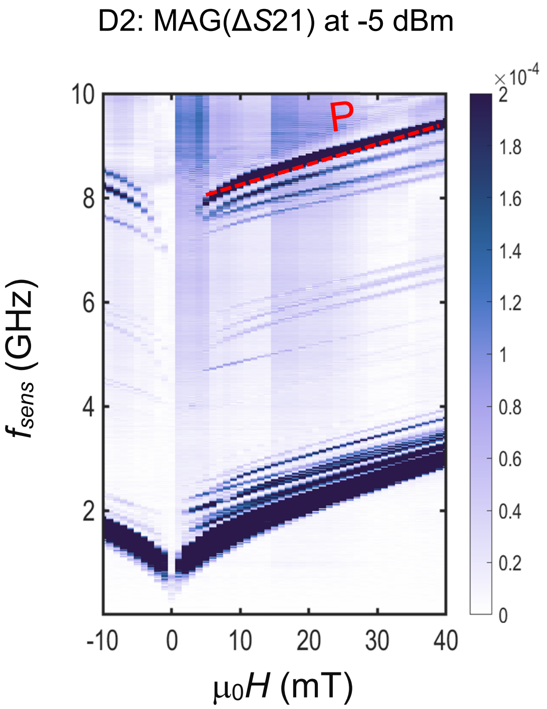

Figure S5: Plot of median subtracted linear magnitude of S21 spectra of device D2 measured at -5 dBm. We do not resolve an AP branch. The onset of the branch P occurs at an applied field of around +5 mT

6. **Calibration of a CPW connected by microwave probes to the vector network analyser**

We performed the calibration of our VNA-based spectroscopy setup and thereby removed the background signal arising from the losses and reflections in RF cables, adapters and microprobes. We performed the SOLT (Source-Open-Load-THRU) calibration at an IF bandwidth of 10 Hz for the frequency range between 10 MHz and 26.5 GHz. The electromagnets providing a bias field during experiments on the probe station were switched OFF during the calibration. We used the Impedance Standard Substrate ISS 101-190C (https://www.formfactor.com/download/iss-map-101-190/?wpdmdl=3159) to calibrate the four modules in the calibration

7. Theoretical model of magnetization reversal via curling

We have considered previous works [1-4] exploring the incoherent magnetization reversal of ferromagnetic wires and Py nanostripes. We use the following formula originally developed for an infinitely long cylinder to estimate the coercive field H_C for a nanostripe array following magnetization curling:

$\mu_0 H_C = \frac{a(1+a)}{\sqrt{a^2+(1+2a)cos^2\theta}} B_{dem}$ ----- **(1)** [2,3] where $a = -1.08(\frac{d_c}{d})^2$.

B_{dem} is the effective demagnetization field acting on the nanostripe. The angle θ is between the long axis of the cylinder and the applied magnetic field. The parameter d_C = 2 x 2.5 x l_{ex} represents the threshold diameter between coherent rotation and reversal by curling. d is the equivalent diameter of a rectangular nanostripe. When $d > d_C$, the magnetization reversal mode is assumed to be curling instead of coherent rotation [2].

To calculate d_C, we considered l_{ex} = 5.7 nm from taken from [5] for permalloy. To calculate d,

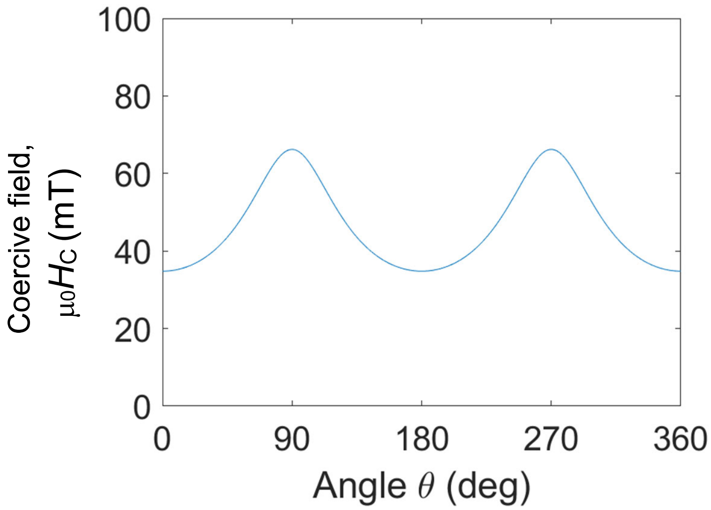

Figure S6: Plot of H_C as a function of field angle modelling magnetization reversal via curling.

we equated the cross-sectional area of a nanostripe in the device D4 (width×height) with the cross-sectional area of a cylinder. An individual nanowire was 100 nm wide and 20 nm high. Hence, the equivalent diameter in D4 amounted to:

$d = 2 \times \sqrt{\frac{100\,nm \times 20\,nm}{\pi}}$ = 50.4 nm,

which was larger than d_C.= 28.5 nm.

B_{dem} = -$N_{eff}\mu_0 M_S$ is determined as -101 mT using the effective demagnetization factor N_{eff} = 0.1 given in the main text of the manuscript and $\mu_0 M_S$ = 1.01 T taken from [5] for permalloy. Figure S6 shows the expected angular dependent coercive field. In the manuscript we compare the calculated coercive field values with the measured data in Fig. 4(f). The stripes with width w = 50 nm and 200 nm fulfill the condition $d > d_C$ for reversal via curling as well.

References:

1. Strijkers, G.; Dalderop, J.; Broeksteeg, M.; Swagten, H.; De Jonge, W. Structure and magnetization of arrays of electrodeposited Co wires in anodic alumina. Journal of Applied Physics 1999, 86, 5141–5145.
2. Han, G. C.; Zong, B. Y.; Luo, P.; Wu, Y. H. Angular dependence of the coercivity and remanence of ferromagnetic nanowire arrays. Journal of Applied Physics 2003, 93, 9202–920
3. Goolaup, S.; Singh, N.; Adeyeye, A.; Ng, V.; Jalil, M. Transition from coherent rotation to curling mode reversal process in ferromagnetic nanowires. The European Physical Journal B-Condensed Matter and Complex Systems 2005, 44, 259–26
4. Pant, B. B. Effect of interstrip gap on the sensitivity of high sensitivity magnetoresistive transducers. Journal of Applied Physics 1996, 79, 6123–612
5. Abo, G. S.; Hong, Y.-K.; Park, J.; Lee, J.; Lee, W.; Choi, B.-C. Definition of Magnetic Exchange Length. IEEE Transactions on Magnetics 2013, 49, 4937–4939
